# Antisense protein of HTLV-2 (APH-2) associates with PML nuclear bodies: molecular determinants and functional implications

**DOI:** 10.1186/1742-4690-11-S1-P100

**Published:** 2014-01-07

**Authors:** Chloé Journo, Jocelyn Turpin, Estelle Douceron, Anaïs Oliva, Renaud Mahieux

**Affiliations:** 1 Oncogenèse Rétrovirale, Equipe Labellisée Ligue Nationale Contre le Cancer, CIRI, INSERM U1111-CNRS UMR5308, Université Lyon 1, Ecole Normale Supérieure de Lyon, LabEx ECOFECT, Lyon, France

## 

Antisens Protein of HTLV-2 (APH-2) was described in 2009. APH-2 mRNA is expressed in vivo in most HTLV-2 carriers. In recent years, several laboratories have searched for similarities and/or differences between APH-2 and the antisens protein of HTLV-1, HBZ. Similarly to HBZ, APH-2 negatively regulates HTLV-2 transcription. However, it does not promote cell proliferation. In vivo, APH-2 localizes in discrete nuclear domains distinct from nucleoli. We therefore characterized APH-2 subcellular localization, in order to decipher the determinants of such localization and to correlate it or not with APH-2 functions. We first identify APH-2-containing nuclear domains as PML nuclear bodies (PML-NB). PML-NB are modulators of a number of cellular processes ranging from transcription regulation to cell proliferation and death. We show that both an in silico-identified nuclear localization signal and the carboxy-terminal LXXLL motif contribute to APH-2 targeting to PML-NB. Covalent modification of APH-2 by SUMO-1 and non-covalent interaction between APH-2 and SUMO-1-modified cellular partners have also been investigated as mechanisms of APH-2 targeting to PML-NB. Our results further demonstrate that APH-2 association with PML-NB is critical for its ability to inhibit viral transcription. This association also leads to a striking decrease in APH-2 stability, suggesting that APH-2 might be active but also targeted to degradation in PML-NB. Finally, we show that APH-2 localization in PML-NB leads to PML-NB clustering and correlates with a decrease in cell proliferation. Altogether, our study sheds new light on the links between the subcellular localization of APH-2 and its cellular functions.

